# Predictors of Poor Outcome of Anti-MDA5-Associated Rapidly Progressive Interstitial Lung Disease in a Chinese Cohort with Dermatomyositis

**DOI:** 10.1155/2020/2024869

**Published:** 2020-11-25

**Authors:** Yuhui Li, Yimin Li, Jian Wu, Miao Miao, Xiaojuan Gao, Wenxin Cai, Miao Shao, Xuewu Zhang, Yan Xu, Lu Cong, Jing He, Xiaolin Sun

**Affiliations:** ^1^Department of Rheumatology and Immunology, Beijing Key Laboratory for Rheumatism and Immune Diagnosis (BZ0135), Peking University People's Hospital, Beijing, China; ^2^Department of Nephrology, Beijing Daxing District People's Hospital, Beijing, China; ^3^Department of Rheumatology, Ningde Hospital, Affiliated Hospital of Fujian Medical University, Ningde, China; ^4^Department of Neurology, People's Hospital of Peking University, Beijing, China

## Abstract

**Objective:**

Antimelanoma differentiation-associated protein 5 (anti-MDA5) autoantibody has been reported in dermatomyositis (DM) to be associated with rapidly progressive interstitial lung disease (RP-ILD). Our study is aimed at determining the clinical characteristics and prognostic factors underpinning anti-MDA5-associated RP-ILD.

**Methods:**

Patients with anti-MDA5-associated DM (aMDA5-DM) were identified at the Peking University People's Hospital. The presence of anti-MDA5 antibody was determined by immunoblotting. Kaplan-Meier, chi-square test, univariate, and multivariate data analyses were used.

**Results:**

Out of 213 patients with DM and clinically amyopathic dermatomyositis (CADM), 20.7% (44/213) of patients were identified as aMDA5-DM. Amongst the aMDA5-DM patients, 63.6% (28/44) were identified as having anti-MDA5-associated RP-ILD. During the follow-up, 32.1% (9/28) of patients with anti-MDA5-associated RP-ILD died of respiratory failure. We identified older age and periungual erythema as two independent risk factors for RP-ILD mortality. Age ≥ 57 years at disease onset was significantly associated with poor survival (*P* = 0.02) in patients with anti-MDA5-associated RP-ILD, while patients with periungual erythema had a better survival rate than those without periungual erythema (*P* < 0.05).

**Conclusions:**

Anti-MDA5-associated RP-ILD is significantly associated with poor survival rates in DM/CADM patients. More effective intervention should be administered to anti-MDA5-associated RP-ILD patients, especially to senior patients and those without periungual erythema.

## 1. Introduction

Dermatomyositis (DM) is a systemic autoimmune disease featuring a characteristic skin rash, proximal muscle weakness, and extramuscular manifestations such as interstitial lung disease (ILD), fever, and arthralgia. ILD is a predictive factor for poor DM/clinically amyopathic dermatomyositis (CADM) outcome [1, 2], and DM patients with rapidly progressive ILD (RP-ILD) are subject to a devastatingly high mortality rate [2, 3].

Myositis specific autoantibodies (MSAs), including anti-TIF-1*γ*, anti-NXP2, anti-Mi-2, anti-MDA5, and anti-SAE, have been found to be associated with DM in the past decade [4]. Different autoantibodies are correlated with different clinical phenotypes. In DM, aMDA5-DM is a specific subtype that has been identified to be associated with RP-ILD [5]. Anti-MDA5 antibody was originally reported on 2005 by Sato et al. [6] in a study of Japanese patients with CADM and RP-ILD. Compared with other DM subtypes, the presence of aMDA5-DM was associated with a lower survival rate due to the high prevalence of RP-ILD [7]. Chen et al. [8] reported that 78.9% of patients with aMDA5-DM developed RP-ILD. Additionally, Chen et al. [9] demonstrated that 85.7% of patients with aMDA5-DM died of RP-ILD. However, due to the heterogeneity of aMDA5-DM, the prevalence of RP-ILD and RP-ILD survival rates are variable [5, 10]. The predictive factors for poor outcome in aMDA5-DM patients with RP-ILD are not well-understood. In the present study, we compared the clinical and laboratory characteristics of patients with aMDA5-DM at the time when they were or were not diagnosed with RP-ILD. We identified initial predictive factors associated with mortality due to respiratory failure in a large anti-MDA5-associated RP-ILD cohort with DM in China.

## 2. Materials and Methods

### 2.1. Study Population

DM/CADM patient data were collected from clinical records of inpatients at the Department of Rheumatology and Immunology in Peking University People's Hospital between July 2014 and October 2019. Patients were included based on a definite DM or CADM diagnosis in accordance with Bohan and Peter or Southeimer's definitions [11, 12]. The study was approved by the ethics committee of Peking University of People's Hospital (2020PH114-01).

The following data from hospital records were gathered at the initial diagnosis of myositis: (1) demographics including age at onset, gender, and disease duration at diagnosis; (2) clinical data including Gottron's sign/papules, skin ulceration, heliotrope rash, neck V-sign, shawl sign, mechanic's hands, periungual erythema, subcutaneous calcinosis, myalgia in proximal limbs, and arthritis; (3) levels of serum muscle enzymes, including creatine kinase (CK, reference value 43-165 U/L), lactate dehydrogenase (LDH, 109-245 U/L), and ferritin (13-150 ng/mL); (4) myositis-specific autoantibodies (antigen including synthetase, NXP2, SAE, Mi-2, TIF-1*γ*, and MDA5), which were identified with immunoblotting according to manufacturers' instructions (Euroimmun, Germany); (5) arterial blood gas analysis, including PaO_2_ and PaCO_2_; (6) pulmonary function tests (PFT) were reported when available, including forced vital capacity (FVC), diffusing capacity for carbon monoxide (DL_CO_), and total lung capacity (TLC); (7) measures from ILD chest images acquired with high-resolution computed tomography (HRCT), including ground-glass attenuation, consolidations, bronchovascular bundle thickening, and evidence of honeycombing or traction bronchiectasis; (8) measures from cytological analysis of bronchoalveolar lavage fluid if performed, including total cell number and ratios of macrophage and lymphocytes. The follow-up period was the duration from diagnosis of myositis to the last visit prior to October 2019 or death.

DM or CADM patients who were positive for anti-MDA5 were defined as aMDA5-DM in accordance with 2019 DM criteria [4]. aMDA5-DM patients were divided into two groups, those with and those without RP-ILD. The RP-ILD group was defined as rapidly progressive dyspnea directly due to ILD, requiring supplementary oxygen, hospitalization, or involving respiratory failure within three months of the ILD diagnosis. The non-RP-ILD group included patients with chronic ILD (C-ILD) and patients without ILD. Exclusion criteria included (1) patients who also had other autoimmune diseases including systemic lupus erythematosus, systemic sclerosis, and rheumatoid arthritis; (2) patients with lung cancer and chronic pulmonary disease including occupational-environmental exposures and chronic obstructive pulmonary disease; (3) patients receiving treatment involving systemic glucocorticoid and immunosuppressant agents before coming to the hospital.

### 2.2. Statistical Analysis

Continuous variables are reported as the mean ± standard deviation, and categorical variables are reported as both count and percentage. Group comparisons between anti-MDA5-associated RP-ILD and non-RP-ILD patients were assessed with Student's *t*-tests or Mann-Whitney *U* tests. Patient outcomes were compared between the anti-MDA5-associated RP-ILD and MDA5-positive non-RP-ILD groups. Survival rate between groups was compared using the Kaplan-Meier curve with the log-rank test. Cox proportional hazards model was used for comparing pulmonary outcomes and survival. First, univariate analysis was done by Cox proportional hazards model, then, the *P* value less than 0.05 and PaO_2_ less than 60 mmHg which means respiratory failure were included in the multivariate analysis by Cox proportional hazards model while forward selection was used in SPSS 23.0.

## 3. Results

### 3.1. Clinical and Laboratory Characteristics of Anti-MDA5-Associated DM

Anti-MDA5 antibody was positive in 20.7% (44/213) of patients with DM/CADM (Table 1). The prevalence of anti-MDA5 antibody in DM and CADM was 7.6% (10/132) and 42% (34/81), respectively. Heliotrope rash, shawl sign, periungual erythema, and skin ulceration were observed to be significantly more common in anti-MDA5-positive patients compared with anti-MDA5-negative patients, with incidence rates of 68.2% vs. 43.2%, 56.8% vs. 27.2%, 29.5% vs. 16.0%, and 38.6% vs. 5.9%, respectively (all *P* < 0.05). On the other hand, the levels of CK (*P* = 0.001) and LDH (*P* = 0.011) were found to be significantly lower in anti-MDA5-positive patients than in anti-MDA5-negative subjects. The prevalence of antiaminoacyl-tRNA synthetase (ARS) antibodies was also significantly lower in anti-MDA5-positive subjects (*P* = 0.001). Anti-MDA5 antibody was significantly associated with RP-ILD, as RP-ILD developed in 63.6% (28/44) of anti-MDA5-positive patients compared with 29.0% (49/169) of anti-MDA5-negative patients (*P* < 0.001). The incidence of death due to ILD in anti-MDA5-positive patients was 20.5% (9/44), significantly higher than that of the anti-MDA5-negative group (4.7%, 8/169) (*P* = 0.002).

### 3.2. Clinical and Laboratory Manifestations of aMDA5-DM Patients with RP-ILD

Out of the 44 patients with aMDA5-DM, 28 had RP-ILD, 8 had C-ILD, and 8 did not have ILD. We compared the clinical and laboratory features between these 3 groups (Table 2). Those with RP-ILD were older (56.0 ± 9.1 years) than those with C-ILD (38.7 ± 13.8 years, *P* < 0.001). There was no significant difference in duration from disease onset to diagnosis, incidence of Gottron's sign/papules, mechanic's hands, heliotrope rash, or skin ulceration between the groups. Moreover, while serum ferritin was higher in patients with RP-ILD compared with those without RP-ILD, there was no significant difference between groups. PaO_2_ was significantly lower in the RP-ILD group than in the C-ILD group (77.02 ± 16.79 vs. 91.65 ± 12.51 mmHg, *P* = 0.042). PFTs variables were significantly decreased in patients with RP-ILD compared with those with C-ILD, including DL_CO_ (60 ± 15.6 vs. 78.2 ± 10.8% predicted, *P* = 0.019) and TLC (80.2 ± 10.5 vs. 100.4 ± 12.6% predicted, *P* = 0.001). Chest HRCT images were available for all patients with ILD. These images revealed a number of features that were more common for patients with RP-ILD compared with those with C-ILD, including ground-glass attenuation (92.9% vs. 37.5%, *P* = 0.003), consolidation (64.3% vs. 12.5%, *P* = 0.016), and bronchovascular bundle thickening (89.3% vs. 50.0%, *P* = 0.030). Analysis of cell composition in BALF demonstrated that patients with RP-ILD had significantly decreased numbers of macrophages compared with patients with C-ILD (42.4 ± 20.1% vs. 61.6 ± 19.0%, *P* = 0.018). The mortality rate due to ILD in patients with RP-ILD was 32.1% (9/28), which was higher than that for patients with C-ILD (0%) or without ILD (0%).

### 3.3. Comparison of Clinical and Laboratory Characteristics between Survived and Nonsurvived Patients with Anti-MDA5-Associated RP-ILD

We divided patients with anti-MDA5-associated RP-ILD into survived and nonsurvived groups (Table 3). Age at disease onset of nonsurvived patients was older than survived patients (62.8 ± 6.2 vs. 53.6 ± 9.2 years, *P* = 0.012). Additionally, the number of peripheral blood lymphocytes was lower in nonsurvived patients compared with survived patients, with average numbers of 0.5 × 10^9^/L and 1.0 × 10^9^/L (*P* = 0.012), respectively. Although serum level of CRP and ferritin were higher in the nonsurvived group than in the survived group, this difference did not reach statistical significance. Likewise, there was no significant difference between the groups in incidences of Gottron's sign/papules, mechanic's hands, heliotrope rash, or skin ulceration. HRCT results were available for all 28 patients. Ground-glass attenuation, consolidation, and traction bronchiectasis were more frequently found in nonsurvived group, but these differences were not statistically significant (Table 3).

### 3.4. Survival Analysis of Patients with RP-ILD in aMDA5-DM

**Figure 1:**
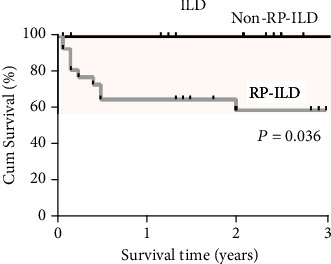
Kaplan-Meier survival curves for RP-ILD and non-RP-ILD groups in aMDA5-DM patients.

**Figure 2:**
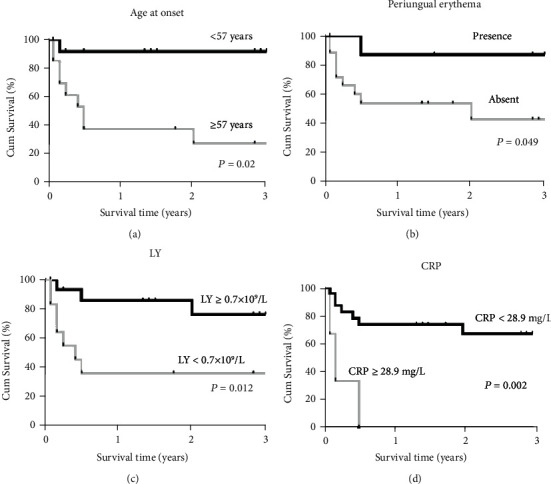
Survival rates between groups stratified by initial predictors of anti-MDA5-associated RP-ILD mortality. Kaplan-Meier survival curves for anti-MDA5-associated RP-ILD: (a) age at onset ≥ 57 years and <57 years; (b) periungual erythema presence versus absent; (c) lymphocyte count ≥ 0.7 × 10^9^/L and <0.7 × 10^9^/L; (d) CRP ≥ 28.9 mg/L and <28.9 mg/L. RP-ILD: rapidly progressive interstitial lung disease; MDA5: melanoma differentiation-associated gene 5; DM: dermatomyositis.

aMDA5-DM patients with RP-ILD had poor survival rates (survival rate: 65.2% after 1 year and 59.2% after 3 years), while no death occurred in patients in the non-RP-ILD group during the 3 years after diagnosis (*P* = 0.036, Figure 1). Results of a survival analysis in the anti-MDA5-associated RP-ILD group are presented in Table 4. Poor prognosis for these patients was associated with an age at onset of ≥ 57 years, having lymphocytes in peripheral blood of less than 0.7 × 10^9^/L and having CRP ≥ 28.9 mg/L. Cox multivariate regression analysis revealed that age at onset ≥ 57 years (HR 18.54, 95% CI 1.51-227.94, and *P* = 0.023) and having periungual erythema (HR 0.08, 95% CI 0.01-0.96, and *P* = 0.046) were independent predictors of mortality due to RP-ILD. The significant predictors from univariate and multivariate survival analyses were then entered into a Kaplan-Meier analysis with log rank (Figure 2). Results indicated that patients whose age was ≥57 years at disease onset had a survival rate of 39% after 1 year and 29.2% after 3 years, while patients who were less than 57 years old at disease onset showed a significantly higher survival rate (92.3% after both 1 and 3 years; *P* = 0.02; Figure 2(a)). Further, patients with periungual erythema at disease onset had better survival (87.5% after both 1 and 3 years) than patients without periungual erythema (54.5% at 1 year and 43.6% at 3 years; *P* = 0.049) (Figure 2(b)). Patients with more peripheral blood lymphocytes (≥0.7 × 10^9^/L) had better survival than those with fewer lymphocytes (<0.7 × 10^9^/L) (*P* = 0.012) (Figure 2(c)). Higher CRP (≥28.9 mg/L) was significantly associated with poorer survival than patients with lower CRP (<28.9 mg/L) (*P* = 0.002) (Figure 2(d)).

## 4. Discussion

In terms of mortality, aMDA5-DM has been identified as a more severe DM subtype than other types of myositis [13, 14]. As a common complication of aMDA5-DM, RP-ILD has been found to be the most important cause of poor survival of this DM subtype [5, 10]. In the current study, we identified initial predictors for the survival of anti-MDA5-associated DM patients with RP-ILD in a patient cohort from China.

The prevalence of anti-MDA5 antibody in our DM/CADM patients was 20.7%, which is consistent with other studies from China (15–20%), but higher than that of patients from Europe and North American (4–7%) [15–17]. Rates of cutaneous manifestations, including ulceration and periungual erythema, observed in patients with anti-MDA5-associated DM were similar to those described previously [18]. Shawl sign was newly revealed to be associated with anti-MDA5-associated DM in this study. The association between heliotrope rash and the MDA5 antibody has been mixed in previous studies. A small-scale retrospective study that included 10 patients with anti-MDA5 by Fiorentino et al. [18] reported that heliotrope rash was not associated with the MDA5 antibody. In contrast, a study on PM/DM patients from Europe reported that heliotrope rash was associated with the MDA5 antibody [15]. Our study revealed a strong association between heliotrope rash and the anti-MDA5 antibody. Relatively low CK and LDH levels observed in our study are consistent with the previous studies [13, 14]. In our cohort, 63.6% of patients with anti-MDA5-associated myositis had RP-ILD, which is consistent with other studies that aMDA5-DM is associated with a high incidence of RP-ILD.

Our results confirmed, in an independent and large Chinese DM/CADM patient cohort, several factors associated with RP-ILD in previous studies, including age at disease onset, decreased lymphocytes in peripheral blood, PaO_2_ levels, baseline DLco and TLC, and evidence of ground-glass attenuation or consolidation in HRCT [10, 19–22]. The association between high baseline concentration of serum ferritin and anti-MDA5-associated RP-ILD has been previously reported [10]; while we found that serum ferritin was higher for patients with RP-ILD, the group difference was not statistically significant, possibly because only a few patients received ferritin tests, leading to insufficient statistical power. Alopecia was reported to be associated with anti-MDA5 antibody in patients with myositis [20]. However, the reverse association between alopecia and anti-MDA5-associated RP-ILD was first revealed in this study. This result suggests that the presence of alopecia might be a protective factor against the development of RP-ILD. The prognostic value of periungual erythema in patients with aMDA5-DM has not been well-examined in previous studies, although a recent study from Japan [22] indicated that periungual erythema is associated with mortality due to ILD. In contrast, another study from Brazil [23] reported that periungual hyperemia was found more common in myositis patients without MDA5 antibody. Our study found that periungual erythema is a protective predictor for anti-MDA5-associated RP-ILD survival and suggest that aMDA5-DM patients with periungual erythema might have better survival.

Mortality due to respiratory failure was observed in 32.1% of aMDA5-DM patients with RP-ILD but not in patients without RP-ILD. Although patients with anti-MDA5 had poorer outcomes, this finding indicated that, in terms of aMDA5-DM, one-third of those patients without RP-ILD still had better survival.

There are several limitations in this study. First, selection bias cannot be ruled out because clinical records were collected from a single center in this retrospective study, and high prevalence of positive aMDA5-DM cannot be avoided since the patients with anti-MDA5 antibody were more likely admitted to hospital. Second, statistical bias cannot be avoided because laboratory tests such as ferritin, CRP, or arterial blood gas analysis were not performed in some patients due to its retrospective design. Therefore, larger population-based multicenter studies are required in the future for more accurate information.

## 5. Conclusions

In conclusion, anti-MDA5-associated RP-ILD is significantly associated with poor outcome in DM patients. Anti-MDA5-associated RP-ILD patients, especially more senior patients and those without periungual erythema, should receive more intensive treatment.

## Figures and Tables

**Table 1:** Comparison of clinical and laboratory characteristics between anti-MDA5-positive and anti-MDA5-negative DM/CADM patients.

Variable	DM/CADM (*n* = 213)		*P* value
	Anti-MDA5-positive group (*n* = 44)	Anti-MDA5-negative group (*n* = 169)	
Age at onset, years	52.38 ± 12.99	51.76 ± 14.66	0.800
DM, no. (%)	10/132 (7.6)	122/132 (92.4)	<0.001
CADM, no. (%)	34/81 (42.0)	47/81 (58.0)	<0.001
Female, no. (%)	31 (70.5)	126 (74.6)	0.582
Fever, no. (%)	17 (38.6)	59 (34.9)	0.646
Gottron's sign/papules, no. (%)	38 (86.4)	129 (76.3)	0.150
Mechanic's hands, no. (%)	23 (52.3)	73 (43.2)	0.281
Heliotrope rash, no. (%)	30 (68.2)	73 (43.2)	0.003
V sign, no. (%)	25 (56.8)	77 (45.6)	0.183
Shawl sign, no. (%)	25 (56.8)	46 (27.2)	<0.001
Subcutaneous calcinosis, no. (%)	0 (0)	7 (4.1)	0.369
Periungual erythema, no. (%)	13 (29.5)	27 (16.0)	0.040
Skin ulceration, no. (%)	17 (38.6)	10 (5.9)	<0.001
Arthritis, no. (%)	24 (54.5)	69 (40.8)	0.102
Myalgia, no. (%)	22 (50.0)	76 (45.0)	0.551
CK, U/L	208.7 ± 559.27	674.49 ± 1327.58	0.001
LDH, U/L	310.56 ± 103.41	374.73 ± 251.14	0.011
CRP level, mg/L	10.59 ± 19.3	13.82 ± 23.39	0.400
Anti-SAE antibody, no. (%)	0 (0)	6 (3.6)	0.349
Anti-Mi-2 antibody, no. (%)	1 (2.3)	8 (4.7)	0.763
Anti-TIF-1*γ* antibody, no. (%)	3 (6.8)	7 (4.1)	0.728
Anti-NXP2 antibody, no. (%)	2 (4.5)	8 (4.7)	1.000
Anti-ARS antibodies, no. (%)	5 (11.4)	63 (37.3)	0.001
ILD, no. (%)	36 (81.8)	128 (75.7)	0.393
RP-ILD, no. (%)	28 (63.6)	49 (29.0)	<0.001
Mortality, no. (%)	9 (20.5)	8 (4.7)	0.002

Categorical data are presented as *n* (percent) of the patients. Continuous data are presented as mean ± standard deviation. DM: dermatomyositis; CADM: clinically amyopathic dermatomyositis; MDA5: melanoma differentiation-associated gene 5; CK: creatine kinase; LDH: lactate dehydrogenase; CRP: C-reactive protein; SAE: small ubiquitin-like modifier enzyme; TIF-1*γ*: translation initiation factor-1*γ*; NXP2: nuclear matrix protein 2; ARS: aminoacyl-tRNA synthetase; ILD: interstitial lung disease.

**Table 2:** Comparison of clinical and laboratory characteristics between the RP-ILD and non-RP-ILD groups in anti-MDA5-positive DM patients.

Variables	aMDA5-DM (*n* = 44)			*P* value	*P* value
	RP-ILD (*n* = 28)	C-ILD (*n* = 8)	Non-ILD (*n* = 8)	RP vs. C-ILD	RP vs. non-ILD
Age at diagnosis, years	56.0 ± 9.1	38.7 ± 13.8	49.4 ± 15.3	<0.001	0.151
Duration from disease onset to diagnosis, months	5.0 ± 6.3	24.8 ± 58.0	5.3 ± 4.6	0.055	0.979
Female, no. (%)	22 (78.6)	5 (62.5)	4 (50)	0.253	0.643
Fever, no. (%)	11 (39.2)	2 (25)	4 (50)	0.892	0.746
Gottron's sign/papules, no. (%)	26 (92.9)	6 (75)	6 (75)	0.436	0.436
Mechanic's hands, no. (%)	14 (50)	3 (37.5)	6 (75)	0.394	0.823
Heliotrope rash, no. (%)	19 (67.9)	6 (75)	5 (62.5)	1.000	1.000
Skin ulceration, no. (%)	13 (46.4)	1 (12.5)	3 (37.5)	0.964	0.185
Periungual erythema, no. (%)	10 (35.7)	2 (25)	1 (12.5)	0.411	0.887
Myalgia, no. (%)	17 (60.7)	3 (37.5)	2 (25)	0.167	0.446
Alopecia, no. (%)	7 (25)	5 (62.5)	5 (62.5)	0.119	0.119
Lymphocytes count, ×10^9^/L	0.9 ± 0.5	1.2 ± 0.5	1.3 ± 0.7	0.081	0.196
CK, U/L	276.0 ± 691.9	127 ± 147.1	53.9 ± 22.5	0.517	0.332
Ferritin (ng/mL)^a^	1417.3 ± 1113.8	54.4 ± 65.5	34.1	0.612	0.142
CRP level, mg/L	13.3 ± 22.9	9.16 ± 12.1	2.7 ± 3.3	0.179	0.598
Arterial blood gas analysis^b^				
PaO_2_, mmHg	77.0 ± 16.8	91.7 ± 12.5	-	0.042	-
PFT (% predicted)^c^				
FVC	79.5 ± 17.1	97.3 ± 17.6	-	0.072	-
DLco	60 ± 15.6	78.2 ± 10.8	-	0.019	-
TLC	80.2 ± 10.5	100.4 ± 12.6	-	0.001	-
HRCT				
Ground-glass attenuation	26 (92.9)	3 (37.5)	-	0.003	-
Consolidation	18 (64.3)	1 (12.5)	-	0.016	-
Traction bronchiectasis	9 (32.1)	0 (0.0)	-	0.160	-
Bronchovascular bundle thickening	25 (89.3)	4 (50.0)	-	0.030	-
Bronchoalveolar lavage^d^					
Total cell number (×105/mL)	0.43 ± 0.50	0.47 ± 0.57	-	0.742	-
Macrophage (%)	42.4 ± 20.1	61.6 ± 19.0	-	0.018	-
Lymphocyte (%)	49.3 ± 22.9	37.3 ± 19.0	-		
Mortality, no. (%)	9 (32.1)	0 (0)	0 (0)	0.076	0.076

Continuous data are expressed as mean ± standard deviation. ^a^8 patients of 28, 20 values missing in RP-ILD group and 7 values missing in the non-ILD group. ^b^1 value missing in the RP-ILD and 4 values missing in the C-ILD. ^c^14 patients of 44, 30 values missing in the RP-ILD group. ^d^22 patients of 44, 22 values missing in the RP-ILD group. RP-ILD: rapidly progressive interstitial lung disease; MDA5: melanoma differentiation-associated gene 5; DM: dermatomyositis; RF: respiratory failure; CK: creatine kinase; LDH: lactate dehydrogenase; PFT: pulmonary function test; FVC: forced vital capacity; DL_CO_: diffusion capacity for carbon monoxide; TLC: total lung capacity.

**Table 3:** Comparison of clinical and laboratory characteristics between survived and nonsurvived patients with anti-MDA5-associated RP-ILD.

Variables	Anti-MDA5-associated RP-ILD (*n* = 28)		*P* value
	Survived group (*n* = 19)	Nonsurvived group (*n* = 9)	
Age at onset, years	53.6 ± 9.2	62.8 ± 6.2	0.012
Female, no. (%)	15 (78.9)	7 (77.8)	1.000
Fever, no. (%)	6 (31.6)	5 (55.6)	0.409
Gottron's sign/papules, no. (%)	18 (94.7)	8 (88.9)	1.000
Mechanic's hands, no. (%)	8 (42.1)	6 (66.7)	0.420
Heliotrope rash, no. (%)	13 (68.4)	6 (66.7)	1.000
Periungual erythema, no. (%)	9 (47.4)	1 (11.1)	0.098
Skin ulceration, no. (%)	8 (42.1)	5 (55.6)	0.689
Alopecia, no. (%)	4 (21.1)	3 (33.3)	0.646
Lymphocytes count, ×10^9^/L	1.0 ± 0.5	0.5 ± 0.3	0.012
CRP level, mg/L	6.4 ± 8.7	27.7 ± 35.5	0.112
Ferritin, ng/mL^a^	806.1 ± 717.8	2028.5 ± 1176.2	0.126
HRCT findings, no. (%)		
Ground-glass attenuation	17 (89.5)	9 (100.0)	1.000
Consolidation	11 (57.9)	7 (77.8)	0.417
Traction bronchiectasis	4 (21.1)	5 (55.6)	0.097
Bronchovascular bundle thickening	17 (89.5)	8 (88.9)	1.000

Categorical data are presented as *n* (percent) of the patients. Continuous data are presented as the mean ± standard deviation. ^a^4 patients of 19, 15 values missing in survived group, and 4 patients of 9, 5 values missing in nonsurvived group. RP-ILD: rapidly progressive interstitial lung disease; MDA5: melanoma differentiation-associated gene 5.

**Table 4:** Survival analysis in anti-MDA5-associated RP-ILD.

	Hazard ratio	95% CI	*P* value
Univariate		
Age at onset ≥ 57 years	11.79	(1.49-93.37)	0.019
CRP ≥ 28.9 mg/L	6.32	(1.56-25.67)	0.010
LY < 0.7 × 10^9^/L	4.80	(1.22-18.92)	0.025
Periungual erythema	0.17	(0.02-1.32)	0.090
PaO_2_ ≤ 60 mmHg	1.78	(0.38-8.40)	0.467
Ferritin	1.00	(1.00-1.00)	0.064
Multivariate		
Age at onset ≥ 57 years	18.54	(1.51-227.94)	0.023
CRP ≥ 28.9 mg/L	6.10	(0.92-40.48)	0.061
LY < 0.7 × 10^9^/L	4.59	(0.75-27.97)	0.098
Periungual erythema	0.08	(0.01-0.96)	0.046
PaO2 ≤ 60 mmHg	9.74	(0.98-97.08)	0.052

Abbreviations: MDA5: melanoma differentiation-associated gene 5; LY: lymphocytes; CRP: C-reactive protein; 95% CI: 95% confidence interval.

## Data Availability

The data that support the findings of this study are available upon request.
